# The emerging roles of deubiquitinases in plant proteostasis

**DOI:** 10.1042/EBC20210060

**Published:** 2022-08-05

**Authors:** Michael J. Skelly

**Affiliations:** Institute of Molecular Plant Sciences, School of Biological Sciences, University of Edinburgh, Edinburgh EH9 3BF, U.K.

**Keywords:** deubiquitinase, plant biology, post translational modification, proteostasis, ubiquitin

## Abstract

Proper regulation of protein homeostasis (proteostasis) is essential for all organisms to survive. A diverse range of post-translational modifications (PTMs) allow precise control of protein abundance, function and cellular localisation. In eukaryotic cells, ubiquitination is a widespread, essential PTM that regulates most, if not all cellular processes. Ubiquitin is added to target proteins via a well-defined enzymatic cascade involving a range of conjugating enzymes and ligases, while its removal is catalysed by a class of enzymes known as deubiquitinases (DUBs). Many human diseases have now been linked to DUB dysfunction, demonstrating the importance of these enzymes in maintaining cellular function. These findings have led to a recent explosion in studying the structure, molecular mechanisms and physiology of DUBs in mammalian systems. Plant DUBs have however remained relatively understudied, with many DUBs identified but their substrates, binding partners and the cellular pathways they regulate only now beginning to emerge. This review focuses on the most recent findings in plant DUB biology, particularly on newly identified DUB substrates and how these offer clues to the wide-ranging roles that DUBs play in the cell. Furthermore, the future outlook on how new technologies in mammalian systems can accelerate the plant DUB field forward is discussed.

## Introduction

To properly grow, reproduce and respond to environmental cues, all organisms must ensure that specific cellular proteins are at optimal levels at the correct times. This requires precise regulation of protein homeostasis (proteostasis), which encompasses the complex and intricate balance between protein synthesis, modification and degradation [1]. Proteostasis is maintained by a wide range of post-translational protein modifications (PTMs) that exponentially increase the magnitude and functionality of proteomes. Ubiquitination, the covalent attachment of the small regulatory protein ubiquitin to target proteins, is an essential and highly tunable PTM involved in most, if not all cellular processes in eukaryotes. Ubiquitin is conjugated via its C-terminus primarily to lysine residues within substrate proteins but also to N-terminal amino acids [2]. Ubiquitination requires the sequential action of three types of enzyme: E1 ubiquitin-activating enzymes, E2 ubiquitin-conjugating enzymes and E3 ubiquitin ligases. Ubiquitin itself contains seven lysine residues that, along with its N-terminal methionine, can be self-modified, facilitating formation of polyubiquitin chains of various linkage types. Different chain types have specific signalling roles and are associated with different cellular processes. For example, K48-linked chains generally label proteins for degradation by the 26S proteasome, a large multiprotein complex providing the major cellular proteolytic activity in eukaryotes [3].

Opposing the actions of the ubiquitin conjugation machinery are a range of deubiquitinase (DUB) enzymes with wide-ranging specificities for different ubiquitin linkages. Deubiquitination of proteins can determine their stability, activity or function, thus entire proteomes can be governed by the activities of specific DUBs [4]. Many human diseases including cancers and neurodegenerative conditions are associated with mutation or aberrant expression of DUBs, demonstrating their importance in regulating physiological processes. Indeed, various DUB inhibitors are emerging as promising therapeutic agents and are now approaching clinical trials [5]. Ubiquitination is especially prevalent in plants, with core components of the ubiquitination machinery accounting for almost 6% of the *Arabidopsis thaliana* (Arabidopsis herein) proteome [6]. While all eukaryotes have relatively few E1 and E2 enzymes, a large variety of E3 ligases and DUBs have been reported. Strikingly, the Arabidopsis genome is predicted to encode for over 1400 E3 ligases compared with ∼600 in humans, indicating a more widespread role for ubiquitin signalling in plants. While ∼100 human DUBs have been identified, the number of plant DUBs is yet to be fully determined. Although some (∼57) DUBs have now been identified in plants [7], their substrates, mechanisms of action and the signalling pathways they regulate are only just beginning to emerge.

**Figure 1:**
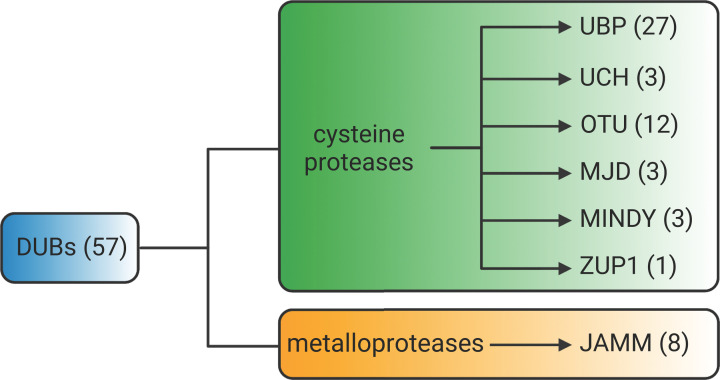
The Arabidopsis DUB families Schematic diagram showing the breakdown of the different DUB families in Arabidopsis. Numbers in parentheses indicate the predicted number of DUBs in each family. Created with BioRender.com.

Plant DUBs have generally been split into five families: UBIQUITIN-SPECIFIC PROTEASES/UBIQUITIN BINDING PROTEASES (USP/UBP), UBIQUITIN C-TERMINAL HYDROLASES (UCH), OVARIAN TUMOR PROTEASES (OTU), MACHADO–JOSEPH DOMAIN PROTEASES (MJD) and JAB1/MPN/MOV34 PROTEASES (JAMM). However, another two families of DUBs have recently been identified in mammals; the MIU-containing novel DUB family (MINDY) [8] and Zinc finger containing Ub Peptidase 1 (ZUP1) [9,10]. These newly identified DUB families are conserved in the Arabidopsis genome, bringing the total number of plant DUB families to seven (Figure 1). Six of the seven DUB families are cysteine proteases, while the JAMM family are metalloproteases. Aside from their catalytic domains, DUBs display a wide range of additional domains that likely determine their substrate specificity and/or incorporation into multiprotein complexes. Recent reviews have comprehensively covered the molecular mechanisms of DUB activity [4,11], and have described in detail the repertoire and phylogeny of plant DUBs [7,12,13]. This minireview will instead discuss only the most recent findings in plant DUB biology, with a focus on DUBs that have known substrates and how they play indispensable roles in proteostasis and cellular signalling.

## The diverse roles of UBP12 and UBP13 – how is substrate specificity determined?

Of all the plant DUB families, the UBPs are by far the most studied with UBP12 and UBP13 perhaps the best characterised. UBP12 and UBP13 share 91% amino acid sequence similarity and appear to be functionally redundant, with single knockout mutants displaying weak phenotypes but null *ubp12 ubp13* double mutants conferring lethality [14]. With this genetic limitation to studying the roles of UBP12/13, most insight has been gained from investigating weak double mutants, transgenic overexpression lines and RNAi-mediated knockdown lines. Most mammalian USPs orthologous to Arabidopsis UBPs have no specificity in terms of ubiquitin linkages that they can cleave [15]. Rather, substrate specificity is thought to be determined by additional protein domains within these DUBs. Similar to their mammalian orthologue USP7, UBP12/13 both have an N-terminal meprin and tumour necrosis factor receptor-associated factor homology (MATH) domain. The MATH domain in human USP7 is required for substrate binding and protein–protein interactions [16] and this also appears to be the case for Arabidopsis UBP12/13. While many substrates have been identified for human USP7, the substrate repertoire for Arabidopsis UBP12/13 has only recently begun to emerge.

The plant hormone jasmonic acid (JA) is a key signalling molecule that regulates a wide range of critical processes including root growth, senescence and defence against both herbivores and necrotrophic pathogens. The transcription factor MYC2 is a master regulator of JA-mediated processes [17] and has been identified as a substrate of UBP12/13 [18]. The N-terminal region of UBP12/13 was shown to be required for binding to MYC2, facilitating its deubiquitination to promote MYC2 stability and downstream JA-mediated signalling [18]. Many DUBs are targeted to their substrates by recognition of, and binding to specific types of ubiquitin modifications rather than direct binding to target proteins. However, UBP12/13 appear to directly recognise their substrate proteins regardless of their ubiquitination status, as binding to MYC2 occurred in pull-down assays using bacterially produced recombinant-purified proteins with no ubiquitin modifications present [18].

UBP12/13 also directly bind and deubiquitinate the ROOT MERISTEM GROWTH FACTOR 1 (RGF1) receptor, RGFR1 [19]. Intriguingly, the present study reported the isolation of a viable *ubp12 ubp13* double mutant, albeit at a very low frequency. These double mutants eventually died at the seedling stage and showed various developmental defects including short roots, attributed in part to severely depleted RGFR1 levels. UBP12/13-mediated deubiquitination of RGFR1 promotes its stability and thereby influences root meristem development via downstream RGF1-mediated signalling. Accordingly, the short root phenotype of *ubp12 ubp13* double mutants was partly alleviated by RGFR1 overexpression, indicating that RGFR1 is a key substrate of UBP12/13 in the context of plant development.

Further examples of direct UBP12/13 substrates have since emerged. These include the transcription factor ORESARA1 (ORE1), which plays a major role in leaf senescence [20]. UBP12/13 directly interact with ORE1 *in vitro* regardless of ORE1 ubiquitination status, providing further evidence of specific UBP12/13-target recognition. ORE1 is deubiquitinated and stabilised by UBP12/13 to promote leaf senescence under nitrogen starvation. Overexpression of UBP12/13 accelerated senescence in wild-type plants but not in *ore1* mutants, while enhanced senescence observed in ORE1 overexpressing lines was further exacerbated by UBP12/13 overexpression. This demonstrates that UBP12/13 act as potent regulators of senescence under nitrogen starvation by maintaining ORE1 protein levels. Another recently identified direct substrate of UBP12/13 is the brassinosteroid (BR) receptor BR INSENSITIVE 1 (BRI1) [21]. In the present study, UBP12/13 were shown to directly bind and deubiquitinate BRI1, promoting its stability. Interestingly, expression of a mutant ubiquitination-deficient BRI1 partly restored the dwarfed growth phenotype of *ubp12* knockdown, *ubp13* knockout (*ubp12i*/*ubp13*) mutant plants. This observation suggests that the developmental defects of *ubp12i*/*ubp13* plants are at least in part due to compromised BRI1 stability and further demonstrate the wide-ranging roles of UBP12/13 in regulating plant developmental processes.

The all above examples represent cases where UBP12/13-mediated deubiquitination directly opposes ubiquitin-mediated degradation of the substrate proteins. However, these are not the only ways in which UBP12/13 DUB activity can regulate proteostasis. The proteins DA1, DAR1 and DAR2 are latent peptidases that are activated by monoubiquitination and cleave a range of plant growth regulators to restrict plant organ growth [22]. A recent study identified both UBP12 and UBP13 as interactors of DA1, DAR1 and DAR2 using mass spectrometry coupled to immunoprecipitation [23]. UBP12/13 deubiquitinate these peptidases rendering them inactive and unable to restrict plant growth. In mutants with down-regulated *UBP12/13* expression, leaf size was smaller due to increased activity of DA1, DAR1 and DAR2. The present study demonstrates the diverse roles of UBP12/13 and together with the other examples outlined above, shows that these DUBs remove a range of ubiquitin modifications, including degradative polyubiquitination and regulatory monoubiquitination.

As well as direct binding to substrates, UBP12/13 can also engage target proteins by their incorporation into multiprotein complexes. Polycomb group proteins (PcG) are a family of proteins that form chromatin remodelling complexes to repress genes in an epigenetic fashion. UBP12 and UBP13 were identified as interacting partners of the plant-specific PcG protein LIKE HETEROCHROMATIN PROTEIN 1 (LHP1) and furthermore, UBP12/13 were shown to have deubiquitination activity towards monoubiquitinated histone H2A [24,25]. UBP12/13 have also been shown to regulate the circadian clock by forming a complex with the E3 ubiquitin ligase photoreceptor ZEITLUPE (ZTL) and the cochaperone protein GIGANTEA (GI) [26]. Although no substrate ubiquitination was analysed in the present study, protein levels of both ZTL and GI were lower in *ubp12* and *ubp13* mutants suggesting that the DUB activity of UBP12/13 stabilises the complex. UBP12/13 did not interact with ZTL but did directly interact with GI through their MATH domains. Therefore, GI serves as a bridge to recruit UBP12/13 to a complex with the E3 ubiquitin ligase ZTL. This raises an intriguing question of why a multiprotein complex would possess both E3 ligase and DUB activity? Surprisingly, the transcription factor TIMING OF CAB2 EXPRESSION 1 (TOC1) that is a known substrate of ZTL also appears to be stabilised by UBP12/13 [26]. Thus, UBP12/13 appear to have multiple roles within this multiprotein complex by stabilising the other components including ZTL but also antagonising ZTL-mediated ubiquitination and degradation of TOC1.

**Figure 2:**
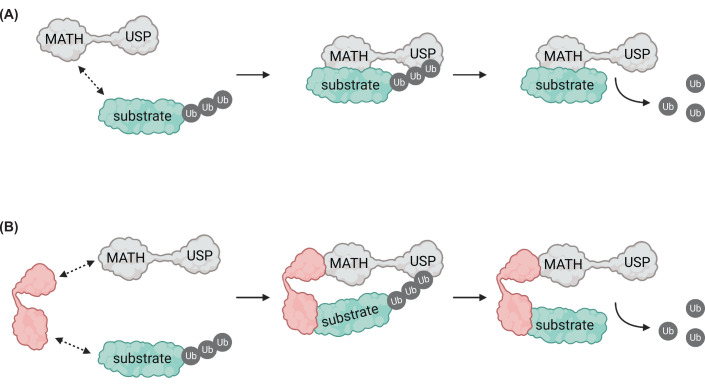
UBP12/13 substrate recruitment (**A**) Some UBP12/13 substrates are directly recruited through binding to the MATH domain, allowing access of the ubiquitinated substrate to the catalytic USP domain. (**B**) Other substrates do not directly bind to UBP12/13 but are recruited via additional adapter proteins. Created with BioRender.com.

Together, the emerging catalogue of UBP12/13 substrates and binding partners highlighted above paints a complex picture of how these DUBs might recognise and deubiquitinate target proteins (Figure 2). The UBP12/13 substrates highlighted above are involved in drastically disparate cellular processes and play critical roles at different times during a plant’s lifecycle or in response to environmental cues. Therefore, mechanisms must be in place to discriminate between substrates that should be deubiquitinated and those that should escape deubiquitination. Further identification of UBP12/13 substrates may reveal common properties of these target proteins that allow their recognition and deubiquitination, whether through direct binding to specific domains of UBP12/13 or to accessory proteins that serve as substrate adapters for these DUBs.

## UBP6 and UBP7 fine-tune plant immunity at the proteasome

Like UBP12/13, Arabidopsis UBP6 and UBP7 are closely related in sequence and appear to act redundantly. Single mutants of either show no obvious phenotype, whereas *ubp6 ubp7* double mutants are compromised in activating immune genes in response to the hormone salicylic acid (SA) [27]. This phenotype was attributed to UBP6/7-mediated deubiquitination of the transcriptional co-activator NONEXPRESSOR OF PATHOGENESIS RELATED GENES 1 (NPR1), which counteracts its proteasomal degradation and promotes its transcriptional activity. UBP6/7 as well as their human and yeast orthologues, USP14 and Ubp6, respectively, are auxiliary components of the 26S proteasome, and only become activated upon proteasome binding [27–29]. Interestingly, yeast Ubp6 can act noncatalytically to inhibit proteasome activity [30]. Together these findings suggest complex cross-regulation between UBP6/7 orthologues and the proteasome in order to control degradation rates of proteasome-bound substrates. The JAMM family DUB, RPN11, is a key component of the 26S proteasome that facilitates protein degradation by removing ubiquitin chains from substrates to allow their access into the proteolytic core. It has been proposed that Ubp6 can delay degradation of ubiquitin-modified proteasome substrates by interfering with RPN11 activity, thereby providing a time window in which Ubp6 can itself deubiquitinate these substrates to spare them from degradation [30,31]. The complexities of Ubp6/USP14-mediated deubiquitination activity at the proteasome were further revealed when it was shown that these DUBs preferentially act on substrates with multiple ubiquitin chains [32].

Unlike UBP12/13 which appear to directly recognise substrate proteins, UBP6/7 are likely to be more promiscuous in their substrate specificity. This is in part because they are inactive unless bound to the proteasome, which precludes surface availability for substrate recognition and a lack of substrate-dedicated protein–protein interaction domains. As well as their catalytic domain, UBP6/7 contain a ubiquitin-like domain (UBL) that is required for binding to the proteasome [33], and a calmodulin-binding domain (CaMBD) [34]. Calmodulins are highly conserved calcium (Ca^2+^) sensors found in all eukaryotes which interact with a broad range of CaMBD-containing proteins to transduce Ca^2+^ signals throughout the cell [35]. Since Ca^2+^ signalling is a prominent feature of plant stress responses [36], UBP6/7 DUB and/or proteasome activity might be regulated by calmodulin binding in response to environmental cues.

With UBP6/7 unlikely to directly bind substrates, what determines if a ubiquitinated protein will be subjected to UBP6/7-mediated deubiquitination at the proteasome? The types and magnitude of ubiquitin modifications on target proteins are the most likely determinants of this. In the case of NPR1, it is likely that this protein is ubiquitin-modified at multiple sites and potentially with different ubiquitin-linkage types. Initial SA-induced ubiquitination by a Cullin-RING Ligase 3 (CRL3) activates the transcriptional activity of NPR1, while ubiquitin chain elongation by Ubiquitin conjugation factor E4 (UBE4) deactivates NPR1 and targets it to the proteasome. Finally, NPR1 undergoes additional polyubiquitination by the proteasome-associated ligases, Ubiquitin Protein Ligases 3 and 4 (UPL3/4) [27,37,38]. Modification by this array of multiple ligases probably leads to a complex mixture of different ubiquitin chains attached to NPR1. Future work to identify the residues that are ubiquitin-modified and the types of ubiquitin chains added to NPR1 will offer more insight into how UBP6/7 might deubiquitinate this substrate. To further add to these complex layers of NPR1 regulation, it appears that UBP6 can also regulate NPR1 activity independently of its DUB activity. Loss of UBP6/7 function in *ubp6 ubp7* null mutants prevents full activation of SA-induced NPR1 target gene expression. The introduction of a *FLAG-UBP6* transgene in the *ubp6/7* double mutant restored NPR1 target gene expression to wild-type levels, while expression of catalytically inactive *FLAG-UBP6(C113S)* restored SA-induced expression of a subset, but not all NPR1 target genes [27]. Therefore, it seems that NPR1 transcriptional activity at different target genes is differentially regulated by either UBP6/7-mediated deubiquitination or UBP6/7-mediated proteasome inhibition. Future identification of other proteins that are deubiquitinated by UBP6/7, and how this affects their degradation and activity, will further our understanding of how dynamic signalling cues are integrated at the proteasome.

## Future perspectives in plant DUB biology

Ubiquitination has been implicated in a wide range of cellular processes in plants, mainly by the identification of key E3 ligases and substrates [39–41]. The emerging roles and substrate repertoires of DUBs highlighted above demonstrate the scale to which these enzymes can influence proteostasis by reversing ubiquitination. Compared with the large efforts focused on understanding plant E3 ligases, DUBs have received much less attention and although many plant DUBs have now been identified, their substrates, mechanisms of action and the signalling pathways they regulate have largely remained elusive. Recent advances in identifying substrates of specific DUBs in model organisms such as Arabidopsis may provide clues towards novel targets for improving plant health and productivity. Indeed, ubiquitination appears to regulate many important agronomic traits in plants including flowering time, seed size and disease resistance [42].

The study of DUBs in mammalian systems has seen astounding advances in the last decade, fuelled by structural studies that have revealed intricate molecular mechanisms of substrate recognition and catalysis [11,15]. Furthermore, advances in proteomics have accelerated DUB identification. While potential plant DUBs have so far been identified using genomic sequence homology methods, DUBs can also be discovered using synthetic ubiquitin suicide probes coupled to mass spectrometry-based identification [43]. These probes are powerful tools that irreversibly label DUB active sites and can reveal whether a given DUB is in an active state, an important consideration since the catalytic activity of many DUBs are under tight post-translational control [15]. Indeed, in mammalian cells, some DUBs show differences between protein abundance and active site labelling, indicating that not all expressed DUBs are enzymatically active [44]. Since these methods have recently identified previously unknown human DUBs [9], applying this approach to plants could potentially identify any atypical plant-specific DUBs that would otherwise be missed using sequence homology models. With the vastly increased number of ubiquitin E3 ligases in plants compared with mammals, it would be unsurprising if plants were to also possess more DUBs than our current estimates predict.

As well as further identification of plant DUBs and their substrates, synthetic engineering of DUBs to direct them to specific neosubstrates could provide a new avenue in which to control the levels of specific cellular proteins. Recently, the technology by which a DUB is fused to a protein-targeting ligand, termed DUB-targeting chimera (DUBTAC), has been used to direct DUB activity towards, and stabilise, proteins involved in human diseases [45,46]. To fully harness the potential of DUBs as tools to manipulate proteostasis in plants, development of plant DUBTACs will undoubtedly play major roles in the future. However, with the plant DUB field still lagging behind mammalian systems in terms of fundamental DUB biology, future work dedicated to the identification, regulation and exploitation of these key enzymes as biotechnological tools is urgently required.

## Summary

- DUBs are key regulators of proteostasis but their research in plants is lacking in comparison with mammalian systems.
- DUBs have diverse substrate specificity and utilise additional domains outside their active sites, and/or recruit adapter proteins, to engage substrates.
- Newly identified DUB substrates in plants include proteins involved in wide-ranging cellular processes such as hormone signalling, development, senescence, circadian clock and immunity.
- Recent technological developments will accelerate future plant DUB research and allow the harnessing of these enzymes as biotechnological tools.

## Open Access

Open access for the present article was enabled by the participation of The University of Edinburgh in an all-inclusive *Read & Publish* agreement with Portland Press and the Biochemical Society under a transformative agreement with JISC.

## Abbreviations

BR: brassinosteroid
BRI1: BR INSENSITIVE 1
CaMBD: calmodulin-binding domain
CRL3: Cullin-RING Ligase 3
DUBs: deubiquitinases
DUBTAC: DUB-targeting chimera
GI: GIGANTEA
JA: jasmonic acid
JAMM: JAB1/MPN/MOV34 PROTEASES
MATH: N-terminal meprin and tumour necrosis factor receptor-associated factor homology
MINDY: MIU-containing novel DUB family
NPR1: NONEXPRESSOR OF PATHOGENESIS RELATED GENES 1
ORE1: ORESARA1
PcG: Polycomb group proteins
PTM: post-translational modifications
RGF1: ROOT MERISTEM GROWTH FACTOR 1
SA: salicylic acid
TOC1: TIMING OF CAB2 EXPRESSION 1
UBE4: Ubiquitin conjugation factor E4
UBL: ubiquitin-like domain
UBP: UBIQUITIN BINDING PROTEASES
UPL3/4: Ubiquitin Protein Ligases 3 and 4
USP: UBIQUITIN-SPECIFIC PROTEASES
ZTL: ZEITLUPE
